# Visualization of Directional Beaming of Weakly Localized Raman from a Random Network of Silicon Nanowires

**DOI:** 10.1002/advs.202100139

**Published:** 2021-05-09

**Authors:** Maria J. Lo Faro, Giovanna Ruello, Antonio A. Leonardi, Dario Morganti, Alessia Irrera, Francesco Priolo, Sylvain Gigan, Giorgio Volpe, Barbara Fazio

**Affiliations:** ^1^ Dipartimento di Fisica e Astronomia Università di Catania via S. Sofia 64 Catania 95123 Italy; ^2^ CNR‐IMM Istituto per la Microelettronica e Microsistemi via Santa Sofia 64 Catania 95123 Italy; ^3^ CNR‐IPCF viale F. Stagno d'Alcontres 37, Faro Superiore Messina 98158 Italy; ^4^ Laboratoire Kastler Brossel ENS‐Université PSL CNRS Sorbonne Université Collège de France 24 rue Lhomond Paris 75005 France; ^5^ Department of Chemistry University College London 20 Gordon Street London WC1H 0AJ UK

**Keywords:** complex optical media, Fourier imaging, Raman scattering, random optical media, silicon nanowires, weak localization of light

## Abstract

Disordered optical media are an emerging class of materials that can strongly scatter light. These materials are useful to investigate light transport phenomena and for applications in imaging, sensing and energy storage. While coherent light can be generated using such materials, its directional emission is typically hampered by their strong scattering nature. Here, the authors directly image Rayleigh scattering, photoluminescence and weakly localized Raman light from a random network of silicon nanowires via real‐space microscopy and Fourier imaging. Direct imaging enables us to gain insight on the light transport mechanisms in the random material, to visualize its weak localization length and to demonstrate out‐of‐plane beaming of the scattered coherent Raman light. The direct visualization of coherent light beaming in such random networks of silicon nanowires offers novel opportunities for fundamental studies of light propagation in disordered media. It also opens venues for the development of next generation optical devices based on disordered structures, such as sensors, light sources, and optical switches.

## Graphical abstract

Coherent light arising from complex optical media is typically nondirectional due to random scattering events. Here, beaming of directional coherent light from a network of random structures is demonstrated and visualized. Generating directional coherent light in random media is a significant step forward toward their integration in next generation optical devices.

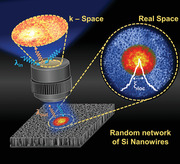

## 1 Introduction

The generation and control of coherent light in optical materials plays a crucial role in different branches of science and technology.^[^ ^1^, ^2^, ^3^ ^]^ It is particularly important for fundamental physics, such as quantum physics and astronomy,^[^ ^4^, ^5^, ^6^ ^]^ as well as for applied physics with applications ranging from communication^[^ ^7^, ^8^ ^]^ to holography,^[^ ^9^ ^]^ to medical imaging.^[^ ^10^, ^11^ ^]^ Directional coherent light signals can be generated by standard cavity‐based lasers^[^ ^12^ ^]^ or by well‐fabricated ordered structures, such as dielectric nanoantennas arrays,^[^ ^13^ ^]^ photonic crystals,^[^ ^14^ ^]^ waveguide systems,^[^ ^15^, ^16^ ^]^ and plasmonic antennas.^[^ ^17^, ^18^ ^]^ Recently, disordered materials have emerged as an inexpensive and easy‐to‐fabricate alternative to these devices for the generation of coherent light,^[^ ^19^, ^20^, ^21^ ^]^ often leading to novel (and, at times, superior) optical performances to those offered by ordered photonic structures.^[^ ^22^, ^23^ ^]^

However, the random morphology of disordered media is generally incompatible with the generation of directional coherent signals. Speckle patterns, arising from the mutual interference of randomly scattered waves, are typical manifestation of this shortcoming of random media as the multiply scattered light leaves the optical material in random directions.^[^ ^3^, ^24^ ^]^ Similarly, in random lasers,^[^ ^25^ ^]^ the radiation amplified by strong multiple scattering is randomly emitted in space, unlike standard lasers where light is amplified along the axis of a cavity. Moreover, due to their multimode operating principle, random lasers typically have no control on spectral characteristics and show unpredictable lasing frequencies.^[^ ^26^ ^]^ So far, a certain degree of control over both modal properties and spatial propagation of coherent signals in disordered materials has been demonstrated via wavefront shaping techniques only.^[^ ^27^, ^28^, ^29^ ^]^

Directional emission of coherent light in disordered optical media is typically a very weak phenomenon, and it can only be observed as a result of averaging over many different realizations of disorder. This is, for example, the case for coherent backscattering (CBS) in random media.^[^ ^30^, ^31^, ^32^ ^]^ In CBS, coherent light waves interfere in reciprocal paths, thus giving rise to an angular emission featuring a cone of coherently enhanced light at the exact backscattering direction. The extraction of the CBS cone from a random optical medium therefore relies on averaging different speckle realizations either by measuring the angular dependence^[^ ^33^ ^]^ or by performing a Fourier transform^[^ ^34^ ^]^ of the light intensity backscattered by the sample.

Here, we report the direct visualization of out‐of‐plane beaming of unimodal Raman light from a random network of quantum‐confined silicon nanowires (Si NWs) without having to resort to complex external light modulation techniques and without the need to average over many different realizations of disorder. By performing real‐space imaging of the inelastic light scattered by the nanostructures, we first visualize directly the strong scattering capability of the random network, where photon diffusion is reduced, and light undergoes a weak localization phenomenon as measured by its localization length. By performing Fourier imaging, we then directly visualize the Raman coherent backscattering cone and demonstrate directional beaming of the weakly localized coherent Raman light from the same random network of silicon nanowires.

## 2 Results

**Figure 1:**
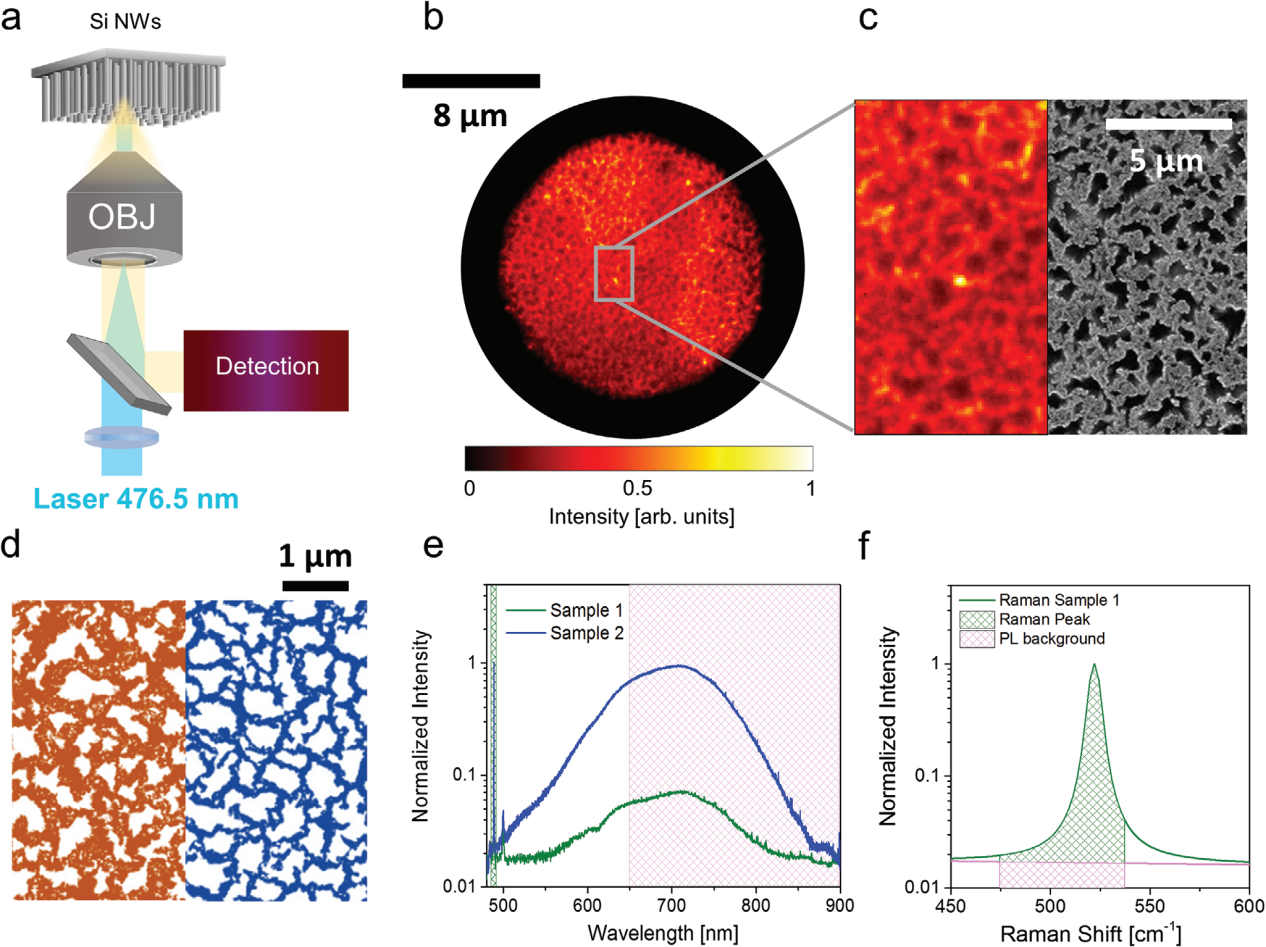
Structural and optical characterization of fractal silicon nanowire arrays. a) Schematics depicting the illumination of the nanowire samples with a laser line at 476.5 nm focused onto the back focal plane of a microscope objective to generate a near‐plane wave at the sample side (100x, N.A. = 0.9). The optical signal from the sample is collected via the same objective and redirected toward the detection system comprising a branch for real‐space imaging, one for spectroscopy and one for angular detection (Section S2 and Figure S2, Supporting Information). b) A typical bright‐field optical microscopy image from a sample of Si nanowires in a random fractal arrangement with 60 ± 2% filling factor (FF) for Sample 1. In order to improve the contrast, the image has been acquired with a reduced field of view as obtained by reducing the aperture of the condenser diaphragm in a Köhler configuration. The background‐subtracted image is normalized to its maximum intensity. c) Comparison between an enlarged detail from b) and a scanning electron micrograph (at low magnification) of the same sample. The two images are reproduced with the same scalebar for direct comparison. d) Scanning electron micrographs of Sample 1 (left) and Sample 2 (right) highlighting the different surface coverages. e) Spectra reporting the sharp Raman signal and the broad photoluminescence (PL) band from both Si nanowire samples (Sample 1 and Sample 2 with stronger photoluminescence). Spectra are normalized to the intensity of the first order Raman peak. The filled areas correspond to the spectral regions used for integrating the Raman (green) and photoluminescence signals (magenta), respectively. In particular, the photoluminescence was integrated in the 650–900 nm range due to the combination of filters used to eliminate the lines corresponding to the residual laser and the Raman contribution (Experimental Section). f) Detail of the Si nanowire 1^st^ order Raman peak, showing the contribution of the integrated Raman signal (green) and photoluminescence background (magenta) based on the employed filters (Experimental Section), respectively. Note that, when excited at 476.5 nm, the photoluminescence background is very low with a negligible integrated intensity (<2%) compared to the integrated intensity of the Raman signal for both samples. Filling factors are expressed as mean ± standard deviation (SD) after averaging over 5 different scanning electron microscopy (SEM) micrographs per sample.

We fabricated disordered arrays of vertically aligned Si nanowires on silicon wafers by metal‐assisted chemical etching (see the Experimental Section).^[^ ^35^ ^]^ The end result is a very dense vertical array of 10^11^–10^12^ nanowires cm^−2^ arranged according to a random fractal planar pattern (Figure S1, Supporting Information).^[^ ^22^ ^]^ The random media are both made of crystalline silicon (c‐Si) in the core of the nanowires, native silicon dioxide in their outer shell and air voids in the gaps separating the nanowires. This fabrication method allows us to obtain nanowires with tunable lengths from 100 nm up to a few tens of µm, and with ultrathin diameters of 7 nm on average.^[^ ^36^ ^]^ Such a small diameter allows for the quantum confinement of charge carriers across the radial direction of the wires, thus leading to a bright room‐temperature photoluminescence in the visible range.^[^ ^37^ ^]^ Moreover, these nanowire arrays support an efficient Raman signal, which presents an asymmetric peak in agreement with the phonon quantum confinement model of Si nanostructures.^[^ ^37^, ^38^ ^]^ Their peculiar optical properties, such as their light trapping, strong multiple scattering and enhanced absorption capabilities, are mainly driven by the 2D random fractal arrangement of these materials^[^ ^22^ ^]^ and are instrumental to the present work. In fact, while very low reflections are expected for this type of Si nanowire arrays, where the incident light resonantly bounces in a random walk across the plane of the 2D fractal, the inelastic signals (due to light‐matter interactions in the sample) propagate in all directions and are very intense, as shown by the spectra in **Figure** **1**. In particular, the high efficiency of the Raman signal is due to the strong multiple scattering of the incident (Rayleigh) radiation within the sample, which, after being efficiently coupled from the direction of incidence to in‐plane propagation by the roughness of the sample silicon substrate at the bottom of the nanowires and by their tips, is resonantly amplified by the underlying planar morphology in the wide range of wavelengths matching its structural heterogeneities (see Section S1 and Figure S1, Supporting Information).^[^ ^39^, ^40^ ^]^ The resonant conditions of the multiply scattered light lead to it being strongly trapped by the random array. This enhances absorption by the nanowires, ultimately leading to the excitation of a really bright photoluminescence visible to the naked eye (Figure 1).^[^ ^22^, ^41^ ^]^ Furthermore, these arrays show a clear structural anisotropy along the vertical direction, while their planar random structure is fully isotropic. Yet, despite this structural anisotropy, these arrays can be considered optically isotropic and so can their scattering properties.^[^ ^42^ ^]^

Such a system of random nanowires therefore offers the unique opportunity to investigate light transport under laser illumination from the comparative study of three main optical signals: i) the Rayleigh (elastic) multiple scattering of the incident radiation, ii) the strong Raman (inelastic) scattering, and iii) the efficient photoluminescence (PL) emission. In particular, these materials have two desirable properties for random media to support coherent Raman effects (i.e., strong multiple scattering and non‐negligible absorption), which arise from the interference between Raman waves travelling reciprocal multiple scattering paths.

In order to investigate the diffusive optical behavior of the Si nanowire arrays, we used a home‐build microscope with three dedicated detection branches for real‐space imaging, for spectroscopy and for angular detection (Figure 1a, Experimental Section; and Figure S2, Supporting Information). In the experiments we focused the external illumination provided by either a white‐light lamp or a laser (*λ* = 476.5 nm) on the back focal plane of a microscope objective (100x, N.A. = 0.9) to produce a nearly collimated beam (full width half maximum, FWHM ≈ 8 µm) on the air‐exposed surface of the nanowires (Figure 1a). This ensured that the laser illuminates the sample as a near‐plane wave, thus impinging on each sample point with the same *k*‐vector (Figure S2, Supporting Information), which is a necessary condition for the correct evaluation of the interference effects coming from the coherent superposition of multiply scattered waves. The sample plane was then imaged on an Electron Multiplying Charge Coupled Device (EM‐CCD) camera to obtain the real‐space morphology of the sample under bright‐field illumination in a standard Köhler configuration to improve image contrast (see the Experimental Section). Figure 1b shows a typical bright‐field image of a silicon nanowire fractal array; as can be seen in the comparison between the optical and the scanning electron images reported in Figure 1c, this bright‐field image, although slightly less resolved than the corresponding scanning electron microscopy (SEM) micrograph due to diffraction, already allows to appreciate the structural features of the fractal sample that are responsible for the optical behavior of the material (see Section S1, Supporting Information).

The direct visualization of Raman coherent effects requires the use of samples with specific structural and optical characteristics to enable a strong scattering regime as well as non‐negligible absorption. These two properties are essential ingredients for supporting a Raman coherent backscattering cone (RCBS).^[^ ^42^ ^]^ In first approximation, in strong scattering regimes, this phenomenon is nonetheless independent from the specific non‐negligible absorption properties of the random sample. Under these constraints, we therefore decided to compare two samples with similar scattering strengths^[^ ^43^ ^]^ but varying non‐negligible absorption free mean paths. Here, the scattering strength is defined as 1/*k*ℓ_t_ (with *k* the wavenumber) and is related to the transport mean free path ℓ_t_, representing the average distance travelled by light across the sample before the direction of its propagation is randomized. This mean free path accounts for the elastically scattered radiation (both Rayleigh and Raman) in the hybrid (Rayleigh‐Raman) multiply scattering paths within the samples. In fact, the Raman light‐wave, once created by an inelastic process (with a very small energy change) propagates by elastic scattering along these paths.

The two selected Si nanowire arrays have similar distributions of their lacunarity (Λ), which probes the sample scattering strength as it quantifies the contrast in refractive index in the fractal array (see Section S1 and Figure S1, Supporting Information). Despite similar scattering strengths, the two samples have different surface coverage defined as the filling factor (FF), which is the ratio between the area filled by the composite material and the total area in a given image (FF = filled area/total area). For the two samples, FF corresponds to 60 ± 2% (Sample 1) and to 45 ± 2% (Sample 2) of the area under study (as shown in Figure 1d) at each observation length‐scale, respectively (see the Experimental Section). These values of FF correspond to a c‐Si fraction of 10% for Sample 1 and 4.5% for Sample 2 within the field of view defined by the microscope objective (corresponding to an area of hundreds of micrometers square). The FF takes into account the absorption properties of the medium since it provides a measure of the amount of material ruling the in‐plane attenuation of the incident light propagating through it. To directly compare the optical absorption properties of the two samples, we can introduce the absorption mean free path ℓ_a_ = (*α*_eff_)^−1^, defined as the distance travelled by light before its intensity is reduced by a factor 1/*e* due to absorption, where $\alpha_{\mathrm{eff}}=\frac{4\pi \kappa_{\mathrm{eff}}}{\lambda }$ is the absorption coefficient for the effective medium with *κ*_eff_ being the imaginary part of the complex effective refractive index (see Section S 1.2 for details, Supporting Information). We estimated that Sample 1 has a shorter ℓ_a_ (about 2.2 times shorter) than Sample 2 due to the higher filling factor (Table 1). As a consequence, we can expect absorption effects in Sample 2 to take place over approximately double the distance than in Sample 1. Aiming to investigate the diffusive optical propagation within the samples, we exploited the bright intensity of the photoluminescence, typically emitted where there is absorption of the excitation radiation from the Si nanowire samples. Having comparable c‐Si volumes for the two samples facilitates this task as it guarantees a similar number of photoluminescence emitting centers in the area illuminated by the laser, so that all differences due to photoluminescence intensity between the two samples can be ascribed to in‐plane propagation. In the fabrication of the samples, we were able to maintain the volume approximately constant by doubling the nanowire length in Sample 2 with respect to Sample 1 (4 µm instead of 2 µm) to compensate for the fact that Sample 2 has about half the c‐Si fraction of Sample 1.

**Table 1:** Structural and optical parameters for the fractal Si nanowire arrays. Values of the filling factor (FF), nanowire length, complex refractive index ${\tilde{n}}_{\mathrm{eff}}$, absorption mean free path ℓ_a_, photoluminescence transverse length *σ*_PL_, Raman transverse localization length *ξ*_loc_ and ratio between *σ*_PL_ and *ξ*_loc_ for Sample 1 and Sample 2. Nanowire lengths and filling factors (FF) are expressed as mean ± standard deviation (SD) as obtained by averaging over five different SEM images per sample. The effective complex refractive index values are obtained by the Bruggeman mixing rule (see Section S1.2, Supporting Information).^[^ ^44^, ^45^ ^]^ Based on the material composition uncertainty, we estimated the error on ${\tilde{n}}_{\mathrm{eff}}$ to be around $\delta {\tilde{n}}_{\mathrm{eff}}=\pm 10\%{\tilde{n}}_{\mathrm{eff}}$. Characteristic optical values for the photoluminescence transverse length and for the localization length were obtained from fitting the average intensity profiles extracted from the analysis of five different images (similar to those in Figure 2) per sample

Si NWs	FF [%]	NWs Length [*μ*m]	${\tilde{n}}_{\mathrm{eff}}=n_{\mathrm{eff}}+i\kappa_{\mathrm{eff}}\;\;\left(\delta {\tilde{n}}_{\mathrm{eff}}=\pm 10\%{\tilde{n}}_{\mathrm{eff}}\right)$	ℓ_a_ [*μ*m]	*σ*_PL_[*μ*m]	*ξ*_loc_ [*μ*m]	*σ*_PL_/*ξ*_loc_
Sample 1	60 ± 2	2 ± 0.5	(at *λ*_exc_ = 476.5 nm) 1.39 + *i*0.010	3.8 ± 0.4	11.5 ± 2.9	4.6 ± 0.7	2.5 ± 1.4
			(at *λ*_Ram_ = 488.6 nm) 1.38 + *i*0.009	4.9 ± 0.5			
			(at *λ*_PL_ = 700 nm) 1.32 + *i*0.001	39 ± 4.0			
Sample 2	45 ± 2	4 ± 0.5	(at *λ*_exc_ = 476.5 nm) 1.17 + *i*0.004	8.6 ± 0.9	16.8 ± 4.4	5.5 ± 1.1	3.1 ± 1.5
			(at *λ*_Ram_ = 488.6 nm) 1.17 + *i*0.004	10.1 ± 1.0			
			(at *λ*_PL_ = 700 nm) 1.14 + *i*0.0006	89.0 ± 10.0			

Beyond the acquisition of real‐space images, our optical setup also allows us to perform the spectral analysis of the nanowire emission through a multimode optical fiber coupled to a spectrometer (Figure S2, Supporting Information). The entire emission spectrum of our Si nanowires under illumination with laser light at 476.5 nm is reported in Figure 1e for both samples. The photoluminescence emission (broad peak in the 500–850 nm range and centered at 700 nm) is really bright, especially for Sample 2. Both spectra also present impressively high and sharp scattered Raman peaks at 488.6 nm due to the 1^st^ order Raman scattering of the Si—Si stretching mode (corresponding to a 520 cm^−1^ Raman shift) (Figure 1f).

**Figure 2:**
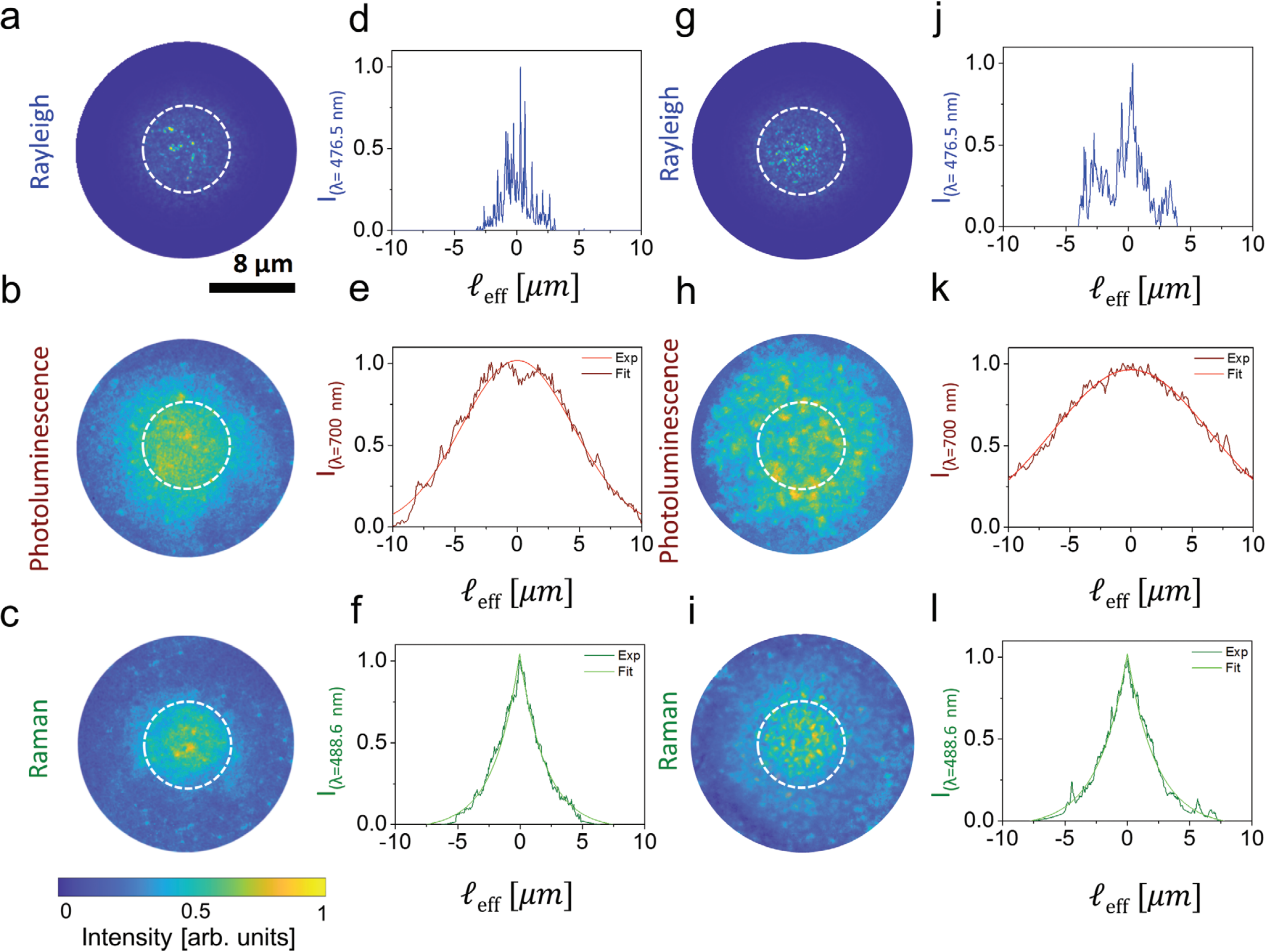
Real‐space optical microscopy of Rayleigh, photoluminescence, and Raman light propagation in the nanowires. a–c) Optical images for the a) Rayleigh, b) photoluminescence, and c) Raman signals and d–f) their relative intensity profiles from Sample 1. g–i) Corresponding optical images and j–l) intensity profiles from Sample 2. The Rayleigh signal has been acquired at 476.5 nm, the photoluminescence in the 650–‐900 nm spectral region, and the Raman signal in the narrow 487–489 nm spectral region. The white dashed circles correspond to the 8 µm area of the sample directly illuminated by the collimated laser beam used for excitation (FWHM ≈8 µm). The reported background‐subtracted optical images a–c,g–i) are normalized to their common maximum of intensity. The intensity profiles for d,j) the Rayleigh signal along a diameter of a,g), e,k) the photoluminescence and f,l) the Raman signals are shown as a function of the effective length ℓ_eff_ = ℓ/*n*_eff_, where ℓ is the distance from the center of the beam scaled for the refractive index of the light in the effective medium *n*_eff_ (Table 1). The photoluminescence and Raman profiles are obtained averaging four intensity profiles per image along the angular coordinate (see the Experimental Section) over five different images and are normalized to their maximum values. These profiles are then fitted with the characteristic e,k) diffusive Gaussian and f,l) localization exponential functions, respectively.

Furthermore, with the correct combination of optical filters (see the Experimental Section and Section S3, Supporting Information), we can image the three signals independently (Rayleigh scattering, photoluminescence emission, or Raman scattering) from the same area in both samples. Typical intensity distributions of the Rayleigh, photoluminescence and Raman signals at the air‐exposed interface of the Si nanowire random arrays are shown in **Figure** **2** for Sample 1 and Sample 2. All images were acquired by using circularly polarized light with a helicity conserving polarization channel (HCC) configuration (Experimental Section and Figure S2, Supporting Information), so to select multiply scattered light only. Indeed, the HCC contribution to the signal intensity has the dual advantage of preserving the coherence effects emerging from multiply scattering paths while intrinsically removing those due to single scattering events or direct reflections from the illumination beam. This choice is mandatory for the Rayleigh scattered radiation, therefore, for consistency, we adopted it as a standard polarization configuration for all signals.

The three optical signals in Figure 2 provide complementary information about the light transport properties of the underlying structure (Figure 2a–f for Sample 1 and Figure 2g–l for Sample 2). First, as shown in Figure 2a,g, the Rayleigh signals of the two samples are reflected and scattered from the Si nanowires as well‐formed speckle patterns within the area of illumination, which are indeed characterized by a typical negative exponential intensity distribution (Figure S4, Supporting Information). Moving away from the illumination area (white dashed line in Figure 2a,g), we however notice a drastic decrease in intensity until the Rayleigh light is totally extinguished, as also confirmed by the intensity profile in Figure 2d,j for Sample 1 and 2, respectively. In fact, both the texturing of the Si nanowire arrays and the roughness of the etched Si substrate interface at the bottom of the sample (intrinsically caused by the nanowire fabrication process) quickly couple the scattered radiation in the sample plane, where the incident light is trapped due to the multiple scattering processes resonantly amplified by the sample 2D fractality. Because of this coupling, light can propagate (minimally affected by reflections or single scattering processes) across the planar structure and out of the excitation area, until fully absorbed by the material. Note that, in order to better evaluate and compare the propagation of signals at different wavelengths through the random array of nanowires, we reported all profiles in Figure 2d–f,j–l as a function of the effective length ℓ_eff_ = ℓ/*n*_eff_ of light propagation from the point of entrance within the effective medium characterized by the wavelength‐dependent *n*_eff_ (Table 1).

Conversely, the images of the vast photoluminescence emission (Figure 2b,h) visibly show a less contrasted speckle intensity distribution over a larger area with respect to the laser spot. Photoluminescence is typically excited where there is absorption of the excitation, and, differently from the Rayleigh case (Figure 2a,g), its image clearly provides an indirect access to the extent of the in‐plane propagation of the excitation light within the material. In fact, each excited Si nanowire radiates photoluminescence in all directions in a wavelength range where the absorption by the nanowires is weaker than at the wavelength of the excitation beam, thus a significant out‐of‐plane photoluminescence can be collected away from the area of excitation. As confirmed by the profiles in Figure 2e,k, being photoluminescence incoherent, its propagation in the random medium follows a diffusive regime characterized by a Gaussian intensity profile, $I=e^{-\ell_{\mathrm{eff}}^{2}/\sigma_{\mathrm{PL}}^{2}}$.^[^ ^46^ ^]^ Here, since this quantity concerns the photoluminescence signal, the effective length ℓ_eff_ is scaled for the effective refractive index calculated at the wavelength where the photoluminescence is the brightest (700 nm), while *σ*_PL_ is the distance along the planar structure (from the center of the exciting beam) at which the photoluminescence intensity reaches 1/*e* ^2^ of its maximum value. Comparing the two samples, we can see how Sample 2 (Figure 2k and Table 1) shows a photoluminescence emission from a wider area (≈6 times the excitation area) and, consequently, a larger *σ*_PL_ than Sample 1 (Figure 2e and Table 1), whose photoluminescence emission already comes from an area ≈ 3 times bigger than the excitation area. Note that in order to measure the emission area of the photoluminescence for the two samples we took into account the FWHM of their photoluminescence intensity distribution as obtained by the relation $\mathrm{FWHM}=\sigma_{\mathrm{PL}}\sqrt{2ln2}$ (13.54 and 19.78 µm for Sample 1 and 2, respectively). This is consistent with a lower attenuation in Sample 2 as testified by its larger ℓ_a_, which, as noted earlier, allows for an extension of the in‐plane propagation of the excitation light over a much larger area than in Sample 1 (Table 1).

Finally, Figure 2c,i shows images of the Raman signal scattered from both samples, which appears more contrasted than the photoluminescence (Figure 2b,h) and more localized than the illumination area. The presence of a weak localization in the Raman scattered light is confirmed by the negative exponential‐like intensity profiles $I=e^{-2|\ell_{\mathrm{eff}}|/\xi_{\mathrm{loc}}}$ in Figure 2f,l, where *ξ*_loc_ is the transverse localization length (i.e., the distance where the diffusion of coherent light in the sample plane is arrested by its disorder).^[^ ^46^, ^47^ ^]^ Here, ℓ_eff_ is obtained by scaling the propagation distance by the effective refractive index of the medium at the wavelength of Raman light (488.6 nm). Both samples have similar *ξ*_loc_ values within the experimental error (Table 1), thus highlighting a similar scattering behavior and confirming the scenario deduced by the analyses of the samples’ lacunarities (Figure S1, Supporting Information). Importantly, the analysis of this real‐space images gives us direct information about the localization length in our random material without the need to average over several realizations of disordered, as typically done in other random media.^[^ ^46^ ^]^ The direct determination of the weak localization length in real space is possible since no Raman speckles are observed, as the acquisition time (tens of seconds in our experiments) is much longer than the coherence time (few picoseconds) of the phonons involved in the Raman process at each scattering site. Thus, the Raman images already are intrinsic averages over a large number of realizations of disorder, being the result of a large number of phonons produced at different times at each scattering site. The Raman signal then provides an advantage when compared to Rayleigh scattering, for which the coherence time is longer, and a localization length can only be extracted when averaging speckle patterns over hundreds different realization of disorder (i.e., over hundreds of images).^[^ ^46^ ^]^

**Figure 3:**
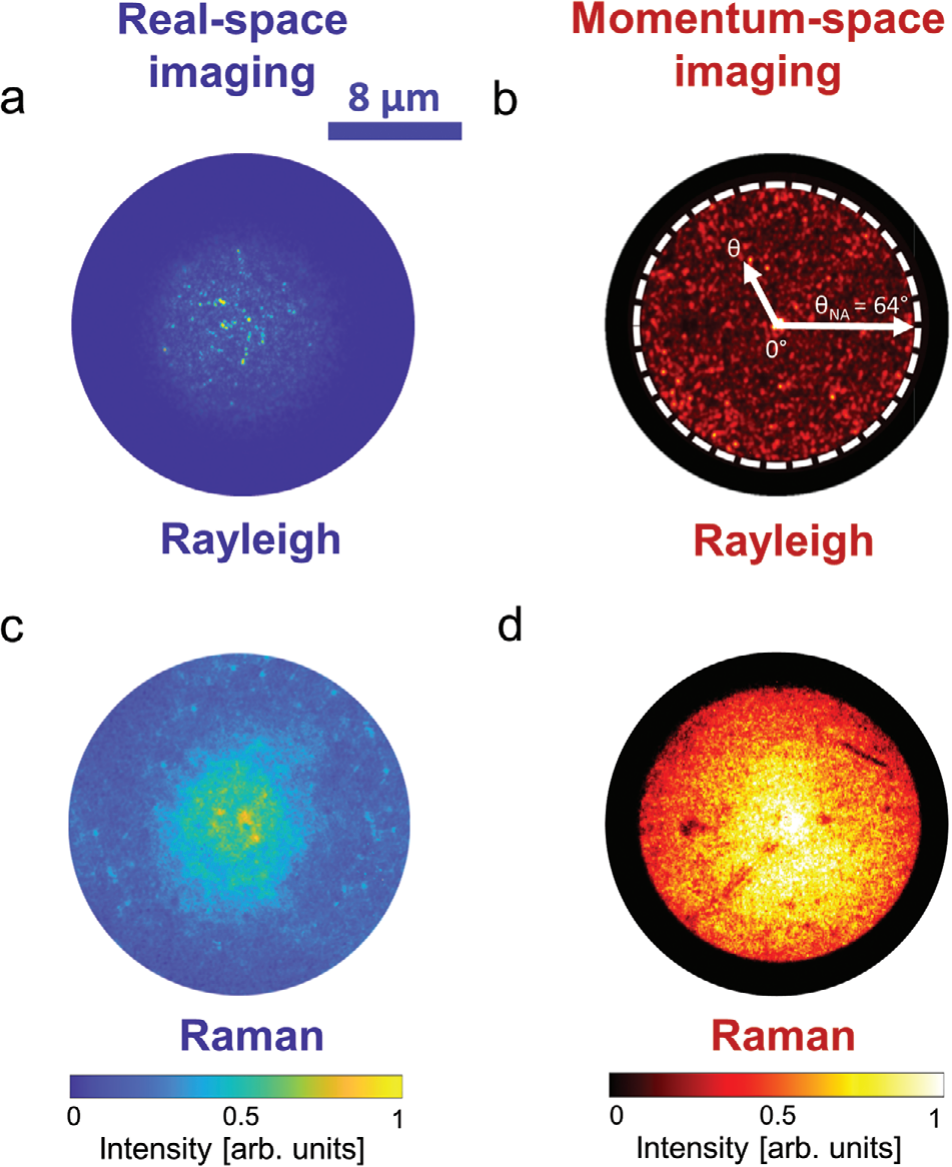
Fourier microscopy and coherent backscattering visualization. Images of a,b) Rayleigh scattering and c,d) Raman scattering in a,c) real space and b,d) Fourier (momentum space) for Sample 1. The white dashed circle in b) highlights the angle corresponding to the numerical aperture (64°) limiting collection through the 100x objective (NA = 0.9) in our setup as depicted from the arrow θ_NA_. The positive angle θ represents the angular cone of acceptance between 0° and θ_NA_ = 64° (i.e., the angle defined by the numerical aperture of the objective). All images have been acquired by exciting the Si nanowires with the 476.5 nm laser line in the helicity conserving polarization (HCC) channel (see the Experimental Section). Both Fourier and real space background‐subtracted images are normalized to the respective common maximum of intensity.

In these random materials the propagating coherent light interferes in reciprocal light paths at the backscattering direction, giving rise to a coherent backscattering cone.^[^ ^43^, ^46^ ^]^ This is also the case for the multiply scattered Raman light.^[^ ^42^ ^]^ Practically, this cone is the Fourier transform of the intensity distribution generated at the output sample surface by all the coherent light paths in the material. This information can therefore be obtained directly by imaging the momentum space of the sample to extract Fourier‐space images. As remarked earlier in the text, unlike the elastic scattering case, the direct imaging of the cone is only possible thanks to the short coherence time of Raman light. As reported in **Figure** **3**, while the real‐space HCC image of the Rayleigh scattered signal (Figure 3a) still presents the expected speckle pattern in Fourier (Figure 3b), the real‐space Raman image (Figure 3c), acquired at the same point in the sample and with the same polarization conditions, does not show a speckle pattern and presents the typical enhanced coherent signature of the nanowire Raman intensity at the exact backscattering direction (0°) in Fourier (Figure 3d).

**Figure 4:**
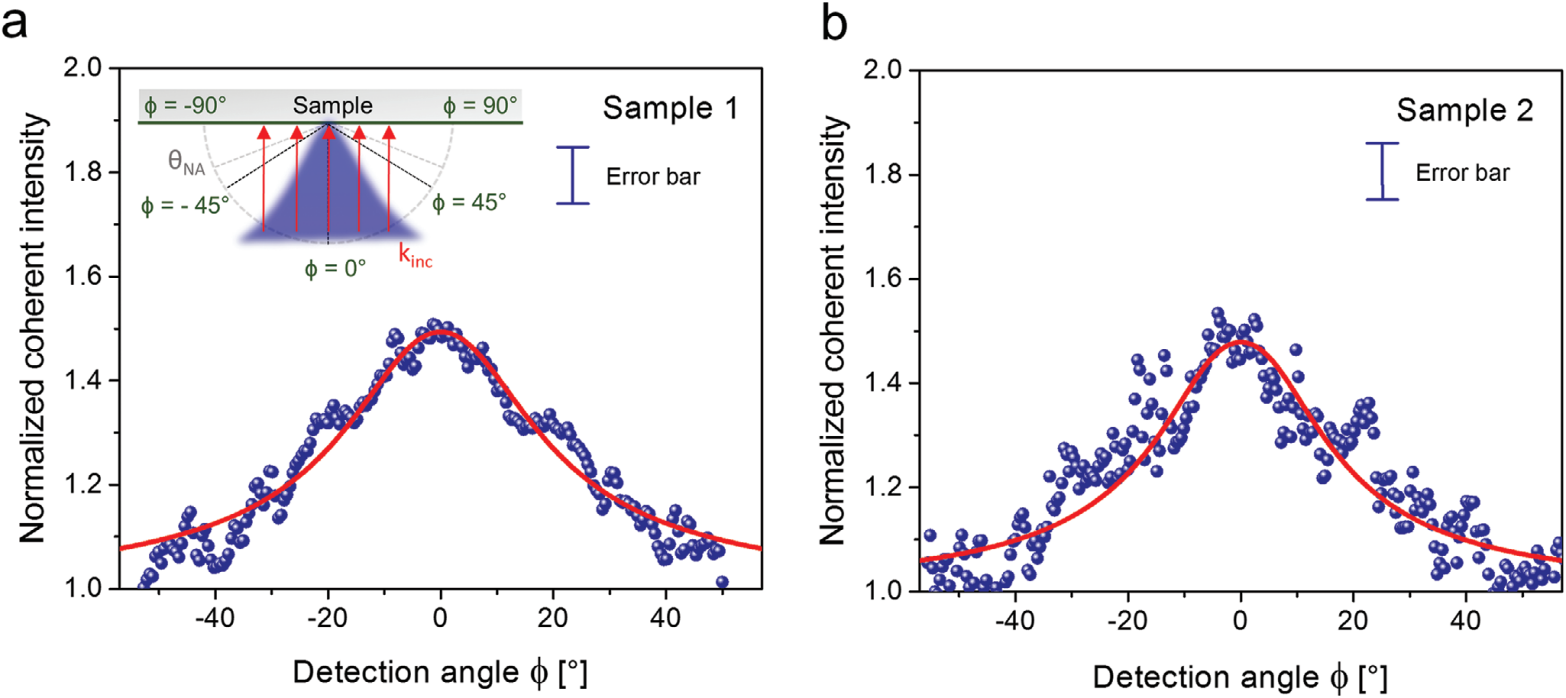
Raman coherent backscattering cone. a,b) Experimental Raman coherent backscattering cones (dots) and fitting curves (solid red lines) for a) Sample 1 and b) Sample 2. The experimental cones were obtained from the ratio between the HCC intensities to the respective incoherent background acquired in the cross‐polarization (Vertical‐Horizontal (VH)) channel (see Section S4, Supporting Information), after normalizing them in order to overlap their values at large angles (at 55°). The sketch in the inset represents both the incident plane‐wave (red arrows) impinging on each sample point with the same *k*‐vector at normal incidence, and the backscattered Raman light directed toward the incident radiation (blue cone). The planar detection angle ϕ is also defined. This angular coordinate is defined with respect to the normal to the sample and is the angular coordinate at which the measurement of the Raman backscattering cone is taken. It varies over 180° (from −90° to 90°), where ϕ = 0° coincides with detecting along the normal to the sample surface. The NA of the objective allows for a detection at an angle varying from −64° to 64°. Error bars on the data points are included in the legend to the graphs and represent the experimental uncertainty of the normalized scattered intensity, taking into account the reliability of both the HCC and VH polarization configurations (see the Experimental Section).

**Figure** **4** shows the experimental Raman coherent backscattering cones (dots) extracted from the momentum‐space images for both Sample 1 and Sample 2 (see Figure S4 and Supporting Section S4, Supporting Information) and reported as a function of the detection angle ϕ. We would like to remark that the structural anisotropy of both samples along the vertical direction does not affect the CBS cone, since the cone is the Fourier transform of the intensity distribution at the output (back‐scattering) surface.^[^ ^49^ ^]^ Moreover, since these random nanowire arrays can be considered optically isotropic,^[^ ^42^ ^]^ the CBS cone can be interpreted using the isotropic scattering model.^[^ ^50^ ^]^ The cones were therefore fitted to the theoretical expression for the Raman coherent backscattering (RCBS) given by, $I\left(\psi \right)=1+\frac{\left(E_{\exp}-1\right)}{\left(E_{\mathrm{Raman}}-1\right)}\frac{\gamma_{C}}{\gamma_{L}}$ where ψ is the angle between the directions of the scattered and the incident radiations (ψ is always 0° at the exact backscattering direction independently of the direction of excitation), *E*_exp_ and *E*_Raman_ represent the experimental (measured) and theoretical enhancement factors of the Raman cone,^[^ ^42^ ^]^ while *γ*_C_(ℓ_a,_ℓ_t,_ℓ_d1_) and *γ*_L_(ℓ_a,_ℓ_t_) are bistatic coefficients or, in other words, the expressions of both coherent and incoherent intensities in terms of the scattered fluxes per solid angle and per unit of probed area.^[^ ^50^, ^51^ ^]^ Both bistatic coefficients are function of the absorption mean free path ℓ_a_ and the transport mean free path ℓ_t_. The coherent contribution to the cone intensity (*γ*_C_) is also function of the dephasing length ℓ_d1_.^[^ ^42^ ^]^ This characteristic length (ℓ_d1_) arises from the dephasing mechanism developed during the mixed random walks of the excitation and of the Raman radiation, and is responsible for the lowering of the Raman cone enhancement with respect to the elastic case for which the theoretical enhancement factor is 2. In the RCBS, while ℓ_t_ accounts for the elastic scattering of Rayleigh and Raman light along hybrid multiple scattering paths, ℓ_d1_ accounts for the inelastic nature of the Raman scattering, is characteristic of the excited Raman mode, and is given by the relation ℓ_d1_ ≈ 2/Δ*k*, where Δ*k* = 2*πn*_eff_|*λ*_exc_ − *λ*_Raman_|/(*λ*_exc_ · *λ*_Raman_) is the magnitude of the exchanged wavevector during the Raman process, and *λ*_exc_ and *λ*_Raman_ are the wavelengths of the excitation and of the Raman scattering, respectively. The experimental enhancement factor *E*_exp_ can be measured from the experimental cone at the exact backscattering angle (ψ = 0°), while the theoretical enhancement *E*_Raman_ is defined as *E*_Raman_ = 1 + *γ*_C_/*γ*_L_.^[^ ^42^ ^]^ In general, the theoretical enhancement differs from the experimental one, both in the elastic and in the inelastic case. Indeed, *E*_exp_ (Raman or Rayleigh) is affected by both residual photons from single scattering events and stray light that introduce possible errors during data acquisition. As a consequence, *E*_exp_ appears reduced with respect to its theoretical value. Moreover, in the Raman case, the ratio *γ*_C_/*γ*_L_ is lower than 1 due to the dephasing mechanism typical of the mixed Rayleigh–Raman random walk;^[^ ^42^ ^]^ as a consequence, *E*_Raman_ is always lower than 2, which is the value expected for the theoretical enhancement factor of the elastic case.

When fitting the RCBS cones in Figure 4, the only fitting parameters are ℓ_t_ and *E*_Raman_, as ℓ_a_ is known (Table 1) and ℓ_d1_ is a fixed value for each sample given by the expression above. The calculated values of ℓ_d1_ are reported in Table 2: although both related to the same Raman mode the small difference between the two samples (ℓ_d1_ is slightly larger for Sample 2) is exclusively due to the difference in filling factor through *n*_eff_. The obtained cone shapes appear large, which points to short transport mean free paths ℓ_t_ and, consequently, to high scattering strengths 1/*k*ℓ_t_. Moreover, within the fitting error, the fitted values of ℓ_t_ are very close to each other, confirming what we previously learnt from the real space images, i.e., that the two samples exhibit a similar behavior in terms of scattering strength due to the similarity of their lacunarity (see Section S1, Supporting Information).

**Table 2:** Raman coherent backscattering parameters for the fractal Si NW arrays. Values of dephasing length ℓ_d1_, transport mean free path ℓ_t_, analytical transverse localization length *ξ*_loc_ and theoretical Raman enhancement *E*_Raman_ for Sample 1 and Sample 2. For both samples, the analytical Raman localization length is calculated from the ℓ_t_ values as ($\xi_{\mathrm{loc}}=\ell_{t}e^{\pi k\ell_{t}/2}$), while the theoretical Raman enhancement factor and the transport mean free path ℓ_t_, were obtained as fitting parameters from fitting the RCBS cone. Their relative errors are evaluated from the confidence interval of the fitting procedure. The values of ℓ_d1_are fixed fitting parameters as obtained by means the relation ℓ_d1_ ≈ 2/Δ*k*, with Δ*k* the exchanged wavevector during the Raman process

Si NWs	ℓ_d1_ [µm]	ℓ_t_ [µm]	*ξ*_loc_ [µm]	*E*_Raman_
Sample 1	4.4 ± 0.1	0.12 ± 0.01	4.1 ± 0.2	1.80 ± 0.02
Sample 2	5.2 ± 0.1	0.14 ± 0.02	4.2 ± 0.2	1.84 ± 0.06

The role played by absorption in this process deserves an in‐depth analysis, as its contribution in RCBS is really subtle. When the absorption mean free path is large (weak absorption), the typical average dephasing between the reciprocal paths can lead to very low Raman cone intensities; conversely, when light absorption in the medium is strong, the average dephasing effect reduces and the cone visibility is not weakened.^[^ ^42^ ^]^ Absorption therefore would become significant enough to modify the cone shape and its enhancement factor only for drastic changes in ℓ_a_ (i.e., by at least one order of magnitude). However, since the absorption mean free path in Sample 2 is only 2.2 times larger than in Sample 1, we do not visibly appreciate its impact on the RCBS cone, and indeed the theoretical enhancement factors *E*_Raman_ for the two samples are found to be relatively close to each other within error bars (Table 2). Although the difference in the absorption mean free path between the two samples is not large enough to imply a consistent difference in terms of the shape of their Raman cones, it is large enough to justify the difference in the photoluminescence emission between the two sample (Figure 1), since it strongly influences the extent of light propagation inside the media (Figure 2).

Starting from the values of transport mean free paths obtained by fitting the RCBS cones, we are also able to calculate the transverse localization lengths $\xi_{\mathrm{loc}}=\ell_{t}e^{\pi k\ell_{t}/2}$ (for the 2D case) for both samples analytically.^[^ ^24^, ^46^ ^]^ The values for *ξ*_loc_ are in good agreement with those previously derived by fitting the intensity distribution profiles in the real‐space Raman images (Figure 2 and Table 1).

**Figure 5:**
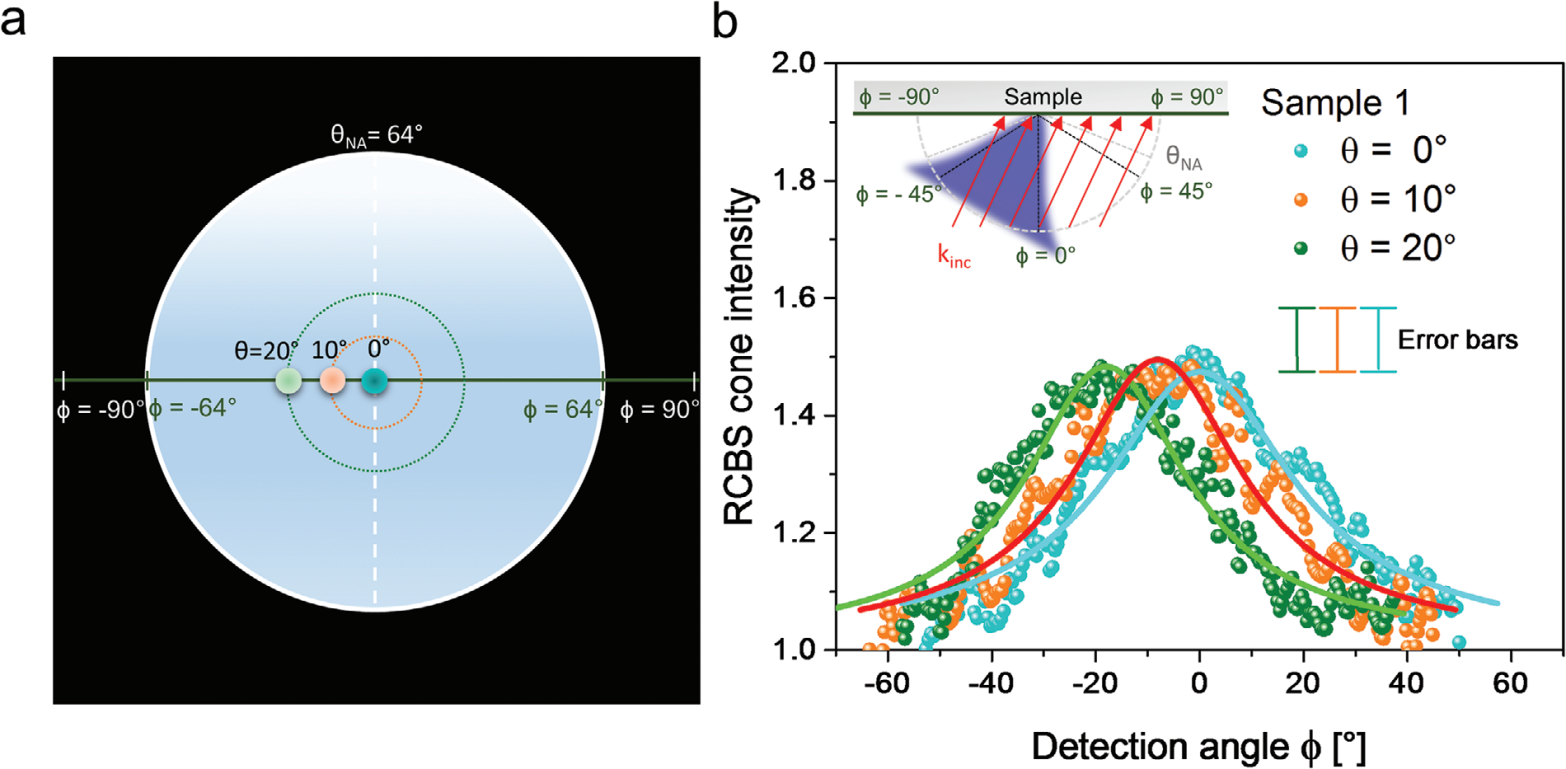
Directional emission of the Raman coherent backscattering cone. a) Scheme depicting the focusing of the laser beam at different points of the back‐focal plane of the objective. These entrance points are defined within different positive incident angles θ within the angular cone of acceptance of the objective, i.e., between 0° and θ_NA_ = 64° (i.e., the angle defined by the numerical aperture of the objective): θ = 0° (blue dot), *θ* = 10° (orange dot), and θ = 20° (green dot). The detection angle ϕ is defined as in Figure 4. b) Normalized Raman coherent backscattering cones (dots) and fitting curves (solid lines) from Sample 1 obtained at the three laser incidences defined in a). Note that, in this case, the exact backscattering angles ψ = 0° coincide each time with the detection angle ϕ = 0°, ϕ = −10°, and ϕ = −20°. The sketch in the inset represents both the incident plane‐wave (red arrows) impinging each sample point with the same *k*‐vector, and the backscattered Raman light directed toward the incident radiation (blue cone). Error bars on the data points are included in the legend to the graphs and represent the experimental uncertainty of the normalized scattered intensity, taking into account the reliability of both the HCC and VH polarization configurations (see the Experimental Section for details).

Finally, the direct visualization of the directional Raman CBS cone from the samples (i.e., without having to average over a large number of disorder configurations) opens up new venues for utilization of random media as devices for the emission of directional coherent light. As shown in **Figure** **5**, when the direction of the incident radiation is changed, the directionality of the coherent Raman emission follows, being directed along the exact backscattering direction on each occasion. This is a typical fingerprint of CBS phenomena. In particular, we imaged different Raman cones as obtained by focusing the excitation beam at different points of the objective back‐focal plane, thus changing its direction of propagation (its *k*‐vector) with respect to normal incidence on the sample surface. As a result, the directionality of the maximum intensity in the Raman cone also shifts accordingly (Figure 5).

## 3 Conclusion

In this work, we have simultaneously visualized the optical signals (Rayleigh, photoluminescence and Raman) coming from random networks of silicon nanowires under laser excitation. These signals were visualized both in real space and in momentum space. The combined direct imaging permitted us to gain insight on the complex scattering and absorption properties behind the investigated materials as well as their interplay beyond what typically allowed by standard angle resolved CBS techniques only. In particular, imaging the photoluminescence emission from the Si nanowires allowed us to directly determine the extent of the in‐plane propagation of the excitation light within the disordered materials. Furthermore, a single coherent Raman image permitted us to directly probe its weak localization length, which would not be possible to extract otherwise from the elastically scattered radiation unless averaging over a large number of different disorder realizations. By imaging the process in momentum space and by taking into account the physics of Raman scattering, we were able to obtain the first direct visualization of the peculiar phenomenon of Raman coherent backscattering. So far, this occurrence has only been observed by means of angle resolved light scattering measurements. Previously, momentum‐space imaging had been employed for patterning the angular distribution of typical Raman bands in 2D materials, such as graphene,^[^ ^52^ ^]^ in order to evaluate their polarization ratio or in plasmonic antennas^[^ ^53^, ^54^, ^55^ ^]^ for estimating their degree of directional emission. In this work, we have further demonstrated the possibility of using Fourier imaging for tuning the directional beaming of coherent Raman light scattered by a random medium. Similar Raman coherent effects can survive in any random medium as long as it strongly scatters light (short transport mean free path) and it has a non‐negligible absorption (short light paths). Our demonstration of light beaming, coupled to the direct visualization of Raman localization, is a convenient way forward to generate coherent and directional light emission from disordered structures, which could be easily implemented in devices without the need for external light modulation techniques.^[^ ^56^, ^57^ ^]^ Coherent Raman radiation offers the additional advantage of fine frequency tuning based on the small energy loss (Stokes) or acquisition (anti‐Stokes) typical of Raman scattering processes. Ultimately, our finding paves the way for the development of next generation inelastic coherent light sources based on random materials with out‐of‐plane directional coherent light control and fine wavelength tuning capabilities.

## 4 Experimental Section

### Fabrication Protocol

Fractal arrays of silicon nanowires were obtained by metal‐assisted chemical etching using a thin percolative gold layer as precursor. After their synthesis, any remaining gold layer is chemically removed. Specifically, n‐type 111‐Si wafers (Siegert Wafer) were first treated with UV‐ozone and then etched in an aqueous solution at 5% of hydrofluoric acid (HF) (49% hydrofluoric acid, Sigma‐Aldrich) in order to remove the native oxide layer on the surface. A 2 nm thick discontinuous gold layer (99.99%, CinquePascal) was then deposited on the clean wafers by using an electron beam evaporator (KM500, Kenosistec) at room temperature. The sample (gold layer + Si substrate) was etched at room temperature in an HF/H_2_O_2_ aqueous solution (5:0.5 m) for the synthesis of the nanowires and then rinsed in potassium iodide (KI) solution (Sigma‐Aldrich) for gold removal. Nanowires obtained by this methodology are made of a crystalline core of silicon (c‐Si) embedded into an outer shell of native silicon dioxide (SiO_2_) ≈ 1–2 nm thick. In this work, two different nanowire samples (Sample 1 and Sample 2) were grown on the same type of Si substrate upon the deposition of a 2 nm thick gold layer obtained at different deposition rates, thus realizing two slightly different fractal geometries. The different deposition rates allow for a distinct distribution of the gold atoms in slightly nonidentical percolative layers, which result in different filling factors and, hence, different effective refractive indices (*n*_eff_). The filling factor (FF) is defined as the ratio between the filled area (in pixels) and the total area (FF = filled pixels/total pixels) in scanning electron microscope images as measured by pixel counting with ImageJ. Sample 1 (FF ≈ 60 ± 2%) and Sample 2 (FF ≈ 45 ± 2%) were obtained with a gold deposition rate of about 0.2^1^ and 0.8 Å s^−1^, respectively. Moreover, the length of the nanowires in the two samples was also varied from 2 µm (Sample 1) to 4 µm (Sample 2) by using different etching times (12 and 20 min, respectively) as shown in Figure S1.

### Structural Characterization

Morphological information about the nanowires was obtained using a scanning electron microscope (SUPRA, Zeiss) equipped with an InLens detector for high‐resolution imaging and with an energy dispersive X‐ray analyzer (EDAX) for analytical measurements. Figure S1a–d (Supporting Information) shows the morphologies of two nanowire arrays as acquired with SEM imaging. The SEM cross‐sections in Figure S1a,b (Supporting Information) display the realization of very dense vertically aligned Si nanowires obtained by the fabrication method described in Section 4.1. The plan‐view SEM micrographs in Figure S1c,d (Supporting Information) show the different surface area coverage characteristic of the two nanowire samples, which is measured by their respective filling factors (FF). From Figure S1c,d (Supporting Information) we measured filling factors of about 60 ± 2% and 45 ± 2% for Sample 1 and Sample 2, respectively. Due to the samples’ fractal properties (in particular, the filling factors scale invariance), at the observation length scale of our experiments, i.e. within the field of view defined by the microscope objective (corresponding to an area of hundreds of micrometers square), we evaluate a c‐Si fraction of 10% for Sample 1 and 4.5% for Sample 2 (see Section S1.2, Supporting Information). Therefore, in order to maintain the c‐Si volume in the two samples approximately constant, we doubled the nanowire length of Sample 2 with respect to Sample 1 (4 µm instead of 2 µm).

### Optical Set Up

All optical images and spectra were obtained with the homemade setup detailed in Figure S2 (Supporting Information). Briefly, the 476.5 nm excitation from an Ar^+^ laser (Spectra Physics 2020) is focused on the back focal plane of a microscope objective (100x, NA = 0.9, Olympus – BX41) by a biconvex lens (L1, Thorlabs) to produce a collimated beam impinging each sample point with nearly the same *k*‐vector. Bright‐field Köhler illumination^[^ ^58^ ^]^ was also implemented by using a white Xenon lamp (Osram) as an alternative source for sample imaging. For image detection, we used a scheme that includes relay optics and a Bertrand lens.^[^ ^54^, ^59^ ^]^ In this configuration, the objective back focal plane is projected through a beam splitter (BS, Thorlabs) and a second biconvex lens (L2, Thorlabs) to form an image of the sample at the image plane (IP). This image is then either recreated by an f‐f‐f'‐f' configuration or Fourier‐transformed in a momentum space image via an f‐f‐2f'‐2f' configuration on an EM‐CCD camera (Andor). The f‐f‐f'‐f' configuration (green dashed lines in Figure S2, Supporting Information) is implemented by using two biconvex lenses (L3 and L4, Thorlabs), while the f‐f‐2f'‐2f' configuration (magenta dashed lines in Figure S2, Supporting Information) is implemented replacing L4 with a biconvex lens (L5, Thorlabs) having half the focal length of L4. Finally, a fiber‐coupled spectrometer (LotOriel 260i) was employed for spectral analysis as depicted in Figure S2 (Supporting Information).

After proper signal filtering, Rayleigh, Raman, and photoluminescence can therefore be observed in real space, in Fourier, or can be spectrally analyzed (see Section S3 of the Supporting Information). A laser interference filter at 473 nm (LD01‐473/10, Semrock) was consistently employed to remove the residual laser line from the detection path.

The acquisition of different optical signals was then performed employing tailored filter sets for detection (see Section S3 and Figure S3, Supporting Information). In particular, Rayleigh light was detected using an optical density filter (OD3, CVI Laser Corporation) to decrease its intensity and avoid saturating the camera. Raman and photoluminescence signals were acquired after removing the Rayleigh line with an edge laser filter (BLP01‐473R‐25, Semrock). For photoluminescence, two additional cooperative long‐pass filters were used (LP550 + LP650, CVI Laser Corporation) for the acquisition of the nanowires’ emission, whose complete range extends from about 500 to 850 nm. After the photoluminescence filter set, the spectral range is restricted to wavelengths above 650 nm. For Raman (Figure S3a,b, Supporting Information), a narrow bandpass filter at 488 ± 1 nm (LL01‐488‐25, Semrock) was used, as this wavelength range corresponds to the 1st order Raman mode at 520 cm^−1^ of the Si—Si bond stretching (observed at 488.6 nm when excited with 476.5 nm). In addition, a short‐pass filter (SP550 from Edmund Optics) was also used to remove the nanowires’ photoluminescence emission in the red region 500–850 nm (Figure S3c,d, Supporting Information). We used the spectrometer to verify the effectiveness of the adopted filter sets to discriminate among the three signals and remove undesired spectral contributions (see Figure 1; and Figure S2, Supporting Information).

All the real‐space images were acquired under a helicity conserving channel (HCC) configuration (Figure S2, Supporting Information) by using in order: 1) a linear polarizer (Thorlabs) to select the input linear polarization, 2) a quarter waveplate (0^th^‐order *λ*/4 at 473 nm, Thorlabs) in order to convert the input linear polarization to circular (note that the output signal from the sample passes a second time through the same quarter waveplate), and 3) another linear polarizer (Thorlabs) in the detection part to select the linear polarization parallel to the input and preserve helicity. This configuration allows us to exclude single scattering events, since they do not preserve helicity, and thus to acquire the coherent signal arising from multiply scattering paths only. Single scattering and reflections are indeed spurious noise for the coherent backscattering experiments and may contribute to a nonisotropic background. Thanks to the HCC configuration, their contribution can be separated from that arising from the portion of multiply scattering paths that preserve helicity and that, instead, contribute to the coherent backscattering.^[^ ^33^ ^]^ Since incoherent contributions with the same helicity can overlap to the coherent ones of the same acquired signal (e.g., in the Raman case), we also need to obtain the incoherent background and normalize the HCC intensity to it in order to obtain only the coherent contribution to the signal intensity. To obtain the incoherent background, we adopted a linear cross‐polarization configuration (VH) simply by removing the quarter waveplate and rotating the output polarizer by 90° (cross‐polarization). We assessed the reliability of the HCC configuration after the microscope objective by measuring the power of the light elastically scattered by a polished silicon flat surface (specular) in the same solid angle around the backscattering. We obtained an HCC fraction over the total circularly polarized scattered light of ≈ 4%. This value quantifies the relative experimental error introduced by the HCC polarization configuration. Similarly, we measured the fraction of power due to the linearly polarized (vertical‐vertical, VV) scattered light over the total linearly polarized scattered light (VV+VH), obtaining a value of ≈ 99%. The relative experimental error for this polarization configuration is then of ≈ 1%. We then measured the HCC fraction over the total circularly polarized scattered light from Sample 1 and 2, obtaining ≈ 35% for Sample 1 and ≈ 20% for Sample 2. This latter sample shows a larger amount of big air voids (larger than 1 µm, as visible in Figure 1d) with respect to Sample 1 and is thus affected by more single scattering events that are efficiently removed by the HCC configuration.

### Statistical Analysis

In this work, we used silicon nanowire samples with a size of 0.5 × 0.5 cm^2^. Data, i.e., nanowire length and sample filling factors are expressed as mean ± standard deviation (SD). The mean values are obtained from the analysis with ImageJ of 5 different scanning electron micrographs for each sample. All images from the CCD were cropped to selected active regions. First, the background was subtracted using ImageJ. Then, the images were plotted and analyzed in MATLAB to extract intensity profiles. For each image, an intensity profile was obtained as an average of 4 distinct profiles (vertical, horizontal, and two diagonal profiles). For the same sample, five different images were acquired at different points; their profiles were extracted as above and used to obtain the average profiles shown in Figure 2. The optical characteristic values reported in Table 1 (i.e., photoluminescence transverse lengths, and localization lengths) were then extracted by fitting these averaged intensity profiles with a Gaussian function (for the photoluminescence signal) or the localization function (for the Raman signal). The optical images for Figures 2, 3; and Figure S5 (Supporting Information) are normalized to the same maximum intensity and are shown using two standard Matlab false‐scale color maps (“parula” for real‐space imaging and “hot” for momentum‐space imaging). The Raman coherent backscattering cone VH and HCC profiles in Figures 4 and 5 are obtained by using the radial profile function in ImageJ and plotted using OriginLab 2019 after being normalized to their intensity at an angle of 55°. The RCBS signal is then obtained from the ratio HCC/VH and analyzed with Wolfram Mathematica by fitting with a finite slab model for the RCBS intensity.^[^ ^50^ ^]^ For both samples, the analytical Raman localization length is calculated from the transport mean free path ℓ_t_ as ($\xi_{\mathrm{loc}}=\ell_{t}e^{\pi k\ell_{t}/2}$), while the theoretical Raman enhancement factor and ℓ_t_ in Table 2, together with their errors, were obtained as fitting parameters from the RCBS fit in Wolfram Mathematica.

## Conflict of Interest

The authors declare no conflict of interest.

## Author Contributions

The manuscript was written through contributions of all authors. All authors have given approval to the final version of the manuscript.

## Supporting information

Supporting Information

## Data Availability

The data that support the findings of this study are available from the corresponding authors upon reasonable request.
