# Supplementary material for: Visualization of Directional Beaming of Weakly Localized Raman from a Random Network of Silicon Nanowires
Source: Adv Sci (Weinh). 2021 May 9;8(14):2100139. doi: 10.1002/advs.202100139 (PMC8292918; doi:10.1002/advs.202100139)
Supplement: Supplementary file 1 — Supporting Information [file ADVS-8-2100139-s001.pdf]

© 2021 Wiley-VCH GmbH

ADVANCED SCIENCE
Open Access

Supporting Information

for *Adv. Sci.,* DOI: 10.1002/advs.202100139

Visualization of directional beaming of weakly localized Raman from a random network of silicon nanowires

Maria J. Lo Faro, Giovanna Ruello, Antonio A. Leonardi, Dario Morganti, Alessia Irrera, Francesco Priolo, Sylvain Gigan, Giorgio Volpe and Barbara Fazio**

Supporting Information

Visualization of directional beaming of weakly localized Raman from a random network of silicon nanowires

Maria J. Lo Faro, Giovanna Ruello, Antonio A. Leonardi, Dario Morganti, Alessia Irrera3, Francesco Priolo 1, Sylvain Gigan 4, Giorgio Volpe 3,5 and Barbara Fazio 3**

Dr. M. J. Lo Faro, Dr. A. A. Leonardi, Dr. D. Morganti, Prof. F. Priolo
Dipartimento di Fisica e Astronomia, Università di Catania, via S. Sofia, 64, 95123 Catania, Italy

Dr. M. J. Lo Faro, Dr. A. A. Leonardi
CNR-IMM, Istituto per la Microelettronica e Microsistemi, via Santa Sofia 64, 95123, Catania, Italy

Dr. M. J. Lo Faro, Dr. G. Ruello, Dr. A. A. Leonardi, Dr. D. Morganti, Dr. A. Irrera, Prof. G. Volpe, Dr. B. Fazio
CNR-IPCF, viale F. Stagno d'Alcontres 37, Faro Superiore, 98158 Messina, Italy

Prof. S. Gigan
Laboratoire Kastler Brossel, ENS-Université PSL, CNRS, Sorbonne Université, Collège de France, 24 rue Lhomond, 75005 Paris, France

Dr. Giorgio Volpe
Department of Chemistry, University College London, 20 Gordon Street, London WC1H 0AJ, UK

Email: g.volpe@ucl.ac.uk, barbara.fazio@cnr.it

Section S1: Structural and optical characterization of fractal silicon nanowires.

Section S1.1: Fractal properties

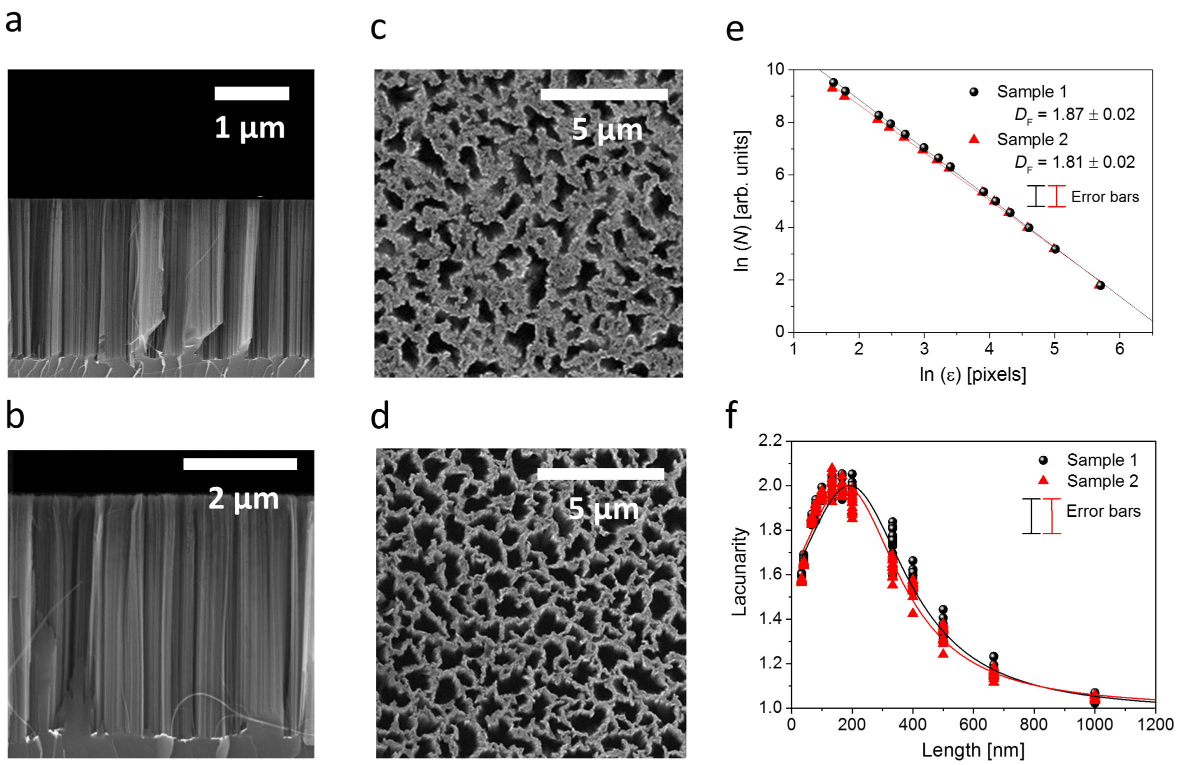

Figure S1: Synthesis and structural characterization of silicon nanowires array. (a-b) Cross-section and (c-d) plan-view scanning electron microscopies of the fractal array of Si nanowires for Sample 1 (a,c) and Sample 2 (b,d), respectively. (e-f) Measured (e) fractal dimension and (f) lacunarity for Sample 1 (black circles) and Sample 2 (red triangles) obtained from box-counting algorithms applied to (c,d) (see Section S1.1). These values are reported as mean ± standard deviation (SD) (error bars). The solid lines (black and red) in (f) are the respective Lorentzian fitting curves. In (f), the length is (nm) = ε (pixel) × 6.67 (nm per pixel), where 6.7 nm is the pixel size for (c,d).

Disordered arrays of vertically aligned Si nanowires show fractal properties.[1,2] Their characteristic parameters of *fractal dimension* (D_F) and *lacunarity* (Λ) were measured as a function of the scale invariance of the system. The fractal dimension D_F measures the complexity of the structure, and shows how the fractal geometry scales as a function of the investigated length scale.[3] The lacunarity Λ is defined as a statistical fluctuation in the distribution (here, the normalized pixel density distribution) of the filled regions of the sample versus its empty regions measured as a function of the investigated length scale. We measured

the fractal parameters of the nanowire structures by using the sliding box-counting algorithm (S_{bcount}) implemented in the FracLac plugin of ImageJ.[4] Briefly, both parameters were measured by counting the number of filled pixels (*N*) in each box of a grid of size ε superimposed onto the SEM plan-view. The process is iterated by varying the grid size (ε) from 37 nm up to 2.2 μm to achieve convergence and repeated for five different initial grid positions in order to acquire a significant statistical ensemble. As this algorithm works on binary images, the SEM plan-view micrographs were first converted into black and white images by ImageJ. The same magnification and image size conditions were used for all the five analyzed images for each sample, for consistency. A 50kX magnification was used to simultaneously probe bigger and smaller dimension ranges, respectively well above and below hundreds of nanometers. The measured fractal dimensions and lacunarities for the two samples are reported in Figure S1e and f, respectively. Figure S1e reports the average number *N* of occupied pixels per box as a function of the box sizes ε, where a linear relation is obtained between these two values in a log-log scale. The fractal dimension D_F is then obtained from the slope of the linear function fitting the pixel density statistics per box size ($D_F = \log N / \log \varepsilon$). The expected D_F for fractal structures is a non-integer value and, as far as 2D fractals are concerned, it is strictly smaller than the Euclidean value of 2. Our analysis confirms that both samples of nanowires are two-dimensional fractals with random geometries having a fractal dimension of 1.87 and 1.81 for Sample 1 and Sample 2, respectively. The lacunarity was calculated as $\Lambda_{\varepsilon,g} = 1 + \sigma^2/\mu^2$, where σ and μ are the standard deviation and the mean of the pixel density distribution calculated for each set of grid dimensions ε and grid positions g used to investigate the image. In total, there are twelve values of lacunarity for each grid dimension ε (the black and red dots in Figure S1d). The lacunarity peak is associated with the maximum heterogeneity in the structure; hence it corresponds to the length scale at which the variations of the effective refractive index are maximized. When light propagates through the medium with an effective wavelength ($\lambda_{\mathrm{eff}} =$

λ/n_{eff}) that matches the length scale at which the lacunarity reaches its peak, the scattering inside the structure is maximized too. Figure S1f shows that, despite a very small shift in the lacunarity peak, the fluctuation of the pixel density is similar for both samples.

S1.2: Estimation of the refractive index

The silicon nanowire fractal arrays show scale invariance of the filling factor (FF)as attested by SEM investigations over three orders of magnification and, therefore, over three length scales.[1] The three length scales extend from a few tens of nanometers up to few micrometers, being the maximal dimension of the observed air voids around 1 μm.[1,3] Considering the fractal properties of our samples, the compositions of the effective media change at each scale of observation, together with their complex refractive indices. Indeed, from lengths of tens of nanometers to lengths of tens of microns the effective material representing the filled space is, from scale to scale, even more affected by a larger number of air voids. As a consequence, the concentration of crystalline silicon (c-Si) decreases as the observation scale increases. We assume that the silicon nanowires are all the same in shape, size and composition, i.e. having all the same crystalline core of 7$\pm$ 2 nm, and the same thickness of native SiO_2 of about 1-2 nm in the outer shell. Therefore, at the largest length scale observed, corresponding to the field of view of the objective microscope (i.e. in an area of hundreds of micrometers square where the biggest air voids are observable, as shown in Figure 1b), the filling factor corresponds to the following compositions for the effective media: ~10% of c-Si, ~10% of SiO_2, 80% of air voids for Sample 1 and ~4.5% of c-Si, ~4.5% of SiO_2, 91% of air voids for Sample 2. Starting from these percentages we can calculate the effective complex refractive indices $\tilde{n}_{\text{eff}} = n_{\text{eff}} + i\kappa_{\text{eff}}$ for the two samples applying the Bruggeman mixing rule for the dielectric permittivity [5,6]. From the imaginary part of the complex refractive indices κ_{eff}, we obtained the effective absorption coefficients for the two samples as $\alpha_{\text{eff}} = \frac{4\pi\kappa_{\text{eff}}}{\lambda}$, which we

used to calculate the absorption mean free paths ℓ_a as shown in Table 1. Although the surface coverage at all length scales has been measured based on the analysis of SEM images, the estimated silicon percentages based on the samples' fractal properties are approximations, since they are obtained assuming that the silicon nanowires are all the same in shape, size and composition. Nonetheless, in the strong scattering regime considered in this work, these approximations do not influence the estimation of the transport mean free path values as these are related to the fluctuations of the refractive index and not to its effective value.

Section S2. Optical set up: acquisition of real-space and Fourier images

In Figure S2 we report the versatile optical set up used in the experiments for the acquisition of both the real-space and the momentum-space images.

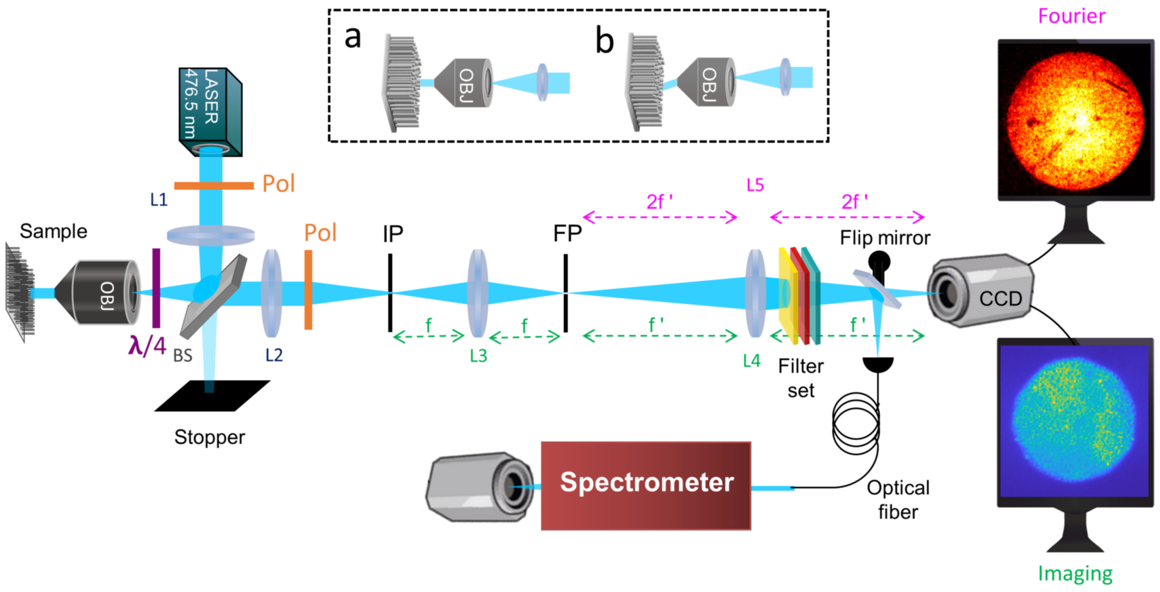

Figure S2: Detailed schematics of the optical setup. The 476.5 nm Ar^+ laser line is focused at the back focal plane of the objective (100x, NA = 0.9) with a lens L1 as to arrive collimated to the sample. The real-space image or Fourier transform of the sample plane are then projected on an EMCCD camera through a set of lenses (L3 and L4 or L3 and L5, respectively). The use of a proper filter set allows us to acquire the optical signal in the desired spectral range as verified with a fiber-coupled spectrometer. A set of two linear polarizers (Pol), and a flip quarter-wave plate (λ/4) were used to select the proper polarization channel for the analysis of the CBS cone. The focal lengths of the lenses in the setup are: f_{L1} = 30 cm; f_{L2} = 30 cm; f_{L3} = 25 cm; f_{L4} = 20 cm; f_{L5} = 10 cm. The top inset shows the configuration used to illuminate the sample with a near-planewave. In this configuration, (a) the laser is focused by a lens on a point of the objective back-focal plane to obtain a

collimated beam on the sample. (b) The orientation of the collimated beam on the sample with respect to the normal incidence is changed by focusing the laser on a point of the back-focal plane far from the optical axis.

Section S3. Acquisition of spectral signals.

We used a fiber-coupled spectrometer from Lot-Oriel coupled with an ultrafast-gated EM-CCD (Andor ICCD DH501-18U-01) in order to discriminate among the three possible signals from the Si nanowires (Rayleigh, photoluminescence and Raman), thus confirming the effectiveness of the adopted filter sets sketched in Figure S3a and c and reported in the Experimental Section of the main text. In Figure S3b and d, we report the spectra corresponding to the first order Raman peak and the photoluminescence band from Si nanowire arrays after the dedicated filter sets, respectively. We remark the critical role of the filters used for Raman imaging (Figure S3a) to remove spurious signals both from the Rayleigh scattering and the broad photoluminescence emission. As shown in Figure S3b, only the sharp Si nanowire Raman peak is present over the entire visible region when the Raman filters are applied. Additionally, in the inset of Figure S3b, the wavelength region close to the laser line at 476.5 nm (blue arrow) is also shown, and no spurious signal from the Rayleigh scattering is present. Similarly, when the PL filters are applied (Figure S3c), only the signal arising from the PL emission of the quantum-confined nanowires can be observed; indeed no signal arises from the wavelength region below the threshold defined by the filter set at 650 nm (Figure S3d). The inset in Figure S3d confirms that no spurious signal is appreciable from the competitive Rayleigh and Raman scattering.

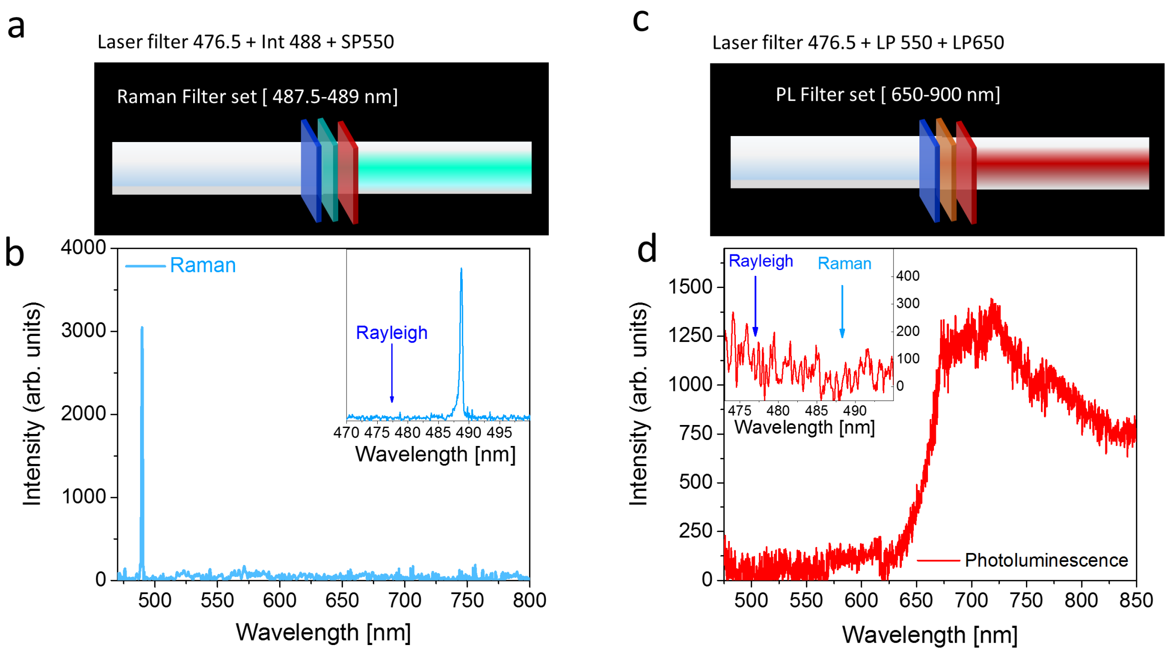

Figure S3: Spectra of the Raman and photoluminescence signals after spectral filtering for Sample 2. We only report the spectra of Sample 2 since it presents more intense Rayleigh and photoluminescence signals than Sample 1. (a) Sketch of the filter set used for the acquisition of the Raman spectrum: Raman light (blue) is collected after passing through 1) an edge laser filter which strongly lowers the laser at 476.5 nm (blue filter), 2) a narrow band-pass filter which reduces the acquisition to a narrow range around 488 ± 1 nm and only lets Raman light through (green filter), and 3) a short-pass filter at 550 nm used to remove the photoluminescence emission above 550 nm (red filter). (b) Raman spectrum from Si nanowires acquired after the dedicated filter set. The inset shows a close-up of the spectral region around the laser wavelength (Rayleigh). (c) Sketch of the filter set used for the acquisition of the photoluminescence spectrum: photoluminescence light (red) is collected after passing through 1) an edge laser filter which strongly lowers the laser at 476.5 nm (blue filter), 2) two long-pass filters with edge at 550 nm (orange and red filters) jointly used to remove both Rayleigh and Raman signals. (d) Photoluminescence spectrum from Si nanowires acquired after the dedicated filter set. The inset shows that both Rayleigh and Raman signals are negligible compared to the photoluminescence intensity fluctuations.

Section S4. Intensity distribution of Rayleigh speckle patterns.

The Rayleigh signals of both Sample 1 and Sample 2 form well-defined speckle patterns. These speckles are characterized by typical negative exponential intensity distributions:[7] $p(I) = \frac{1}{\langle I \rangle} e^{-I/\langle I \rangle}$, as shown in Figure S4.

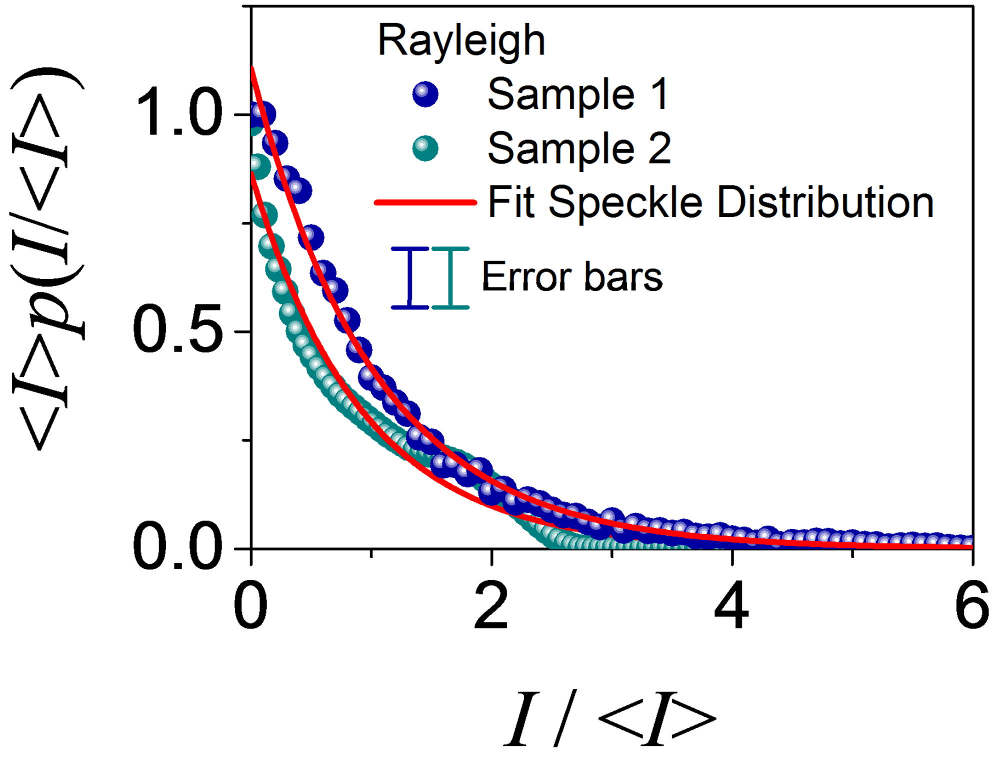

Figure S4: Speckle pattern intensity distributions. Intensities distributions of Rayleigh speckle patterns for Sample 1 (blue dots) and for Sample 2 (green dots). Data are obtained plotting the intensity distribution as function of intensity values after averaging over 5 different images. Data are reported as mean ± standard deviation (SD) (error bars). The red lines represent the negative exponential fitting curves.

Section S5. Analysis of the Raman Coherent Backscattering Cone

In Figure S5 we show the momentum-space images of the Raman signal for the helicity conserving polarization channel (HCC, FigureS5a,c) and for the linear cross-polarization channel (VH, FigureS5b,d) for both Sample 1 (FigureS5a-b) and Sample 2 (FigureS5c-d). In order to extract the cone shape, we first obtained an average intensity profile for each image in the HCC and VH channels. In particular, by using the plot profile function from ImageJ, we averaged the profiles obtained along the diameters in the *x*-axis, the *y*-axis, and the two main diagonals of the circle defined by the numerical aperture of the objective. We then scaled the average intensity profiles for the HCC and VH channels in order to overlap their values at

large angles (around 55°). Finally, we normalized the two profiles to one another (I_{HCC}/I_{VH}) in order to achieve the enhanced Raman coherent backscattering cone (RCBS) for each sample as shown in Figure4 and 5b.

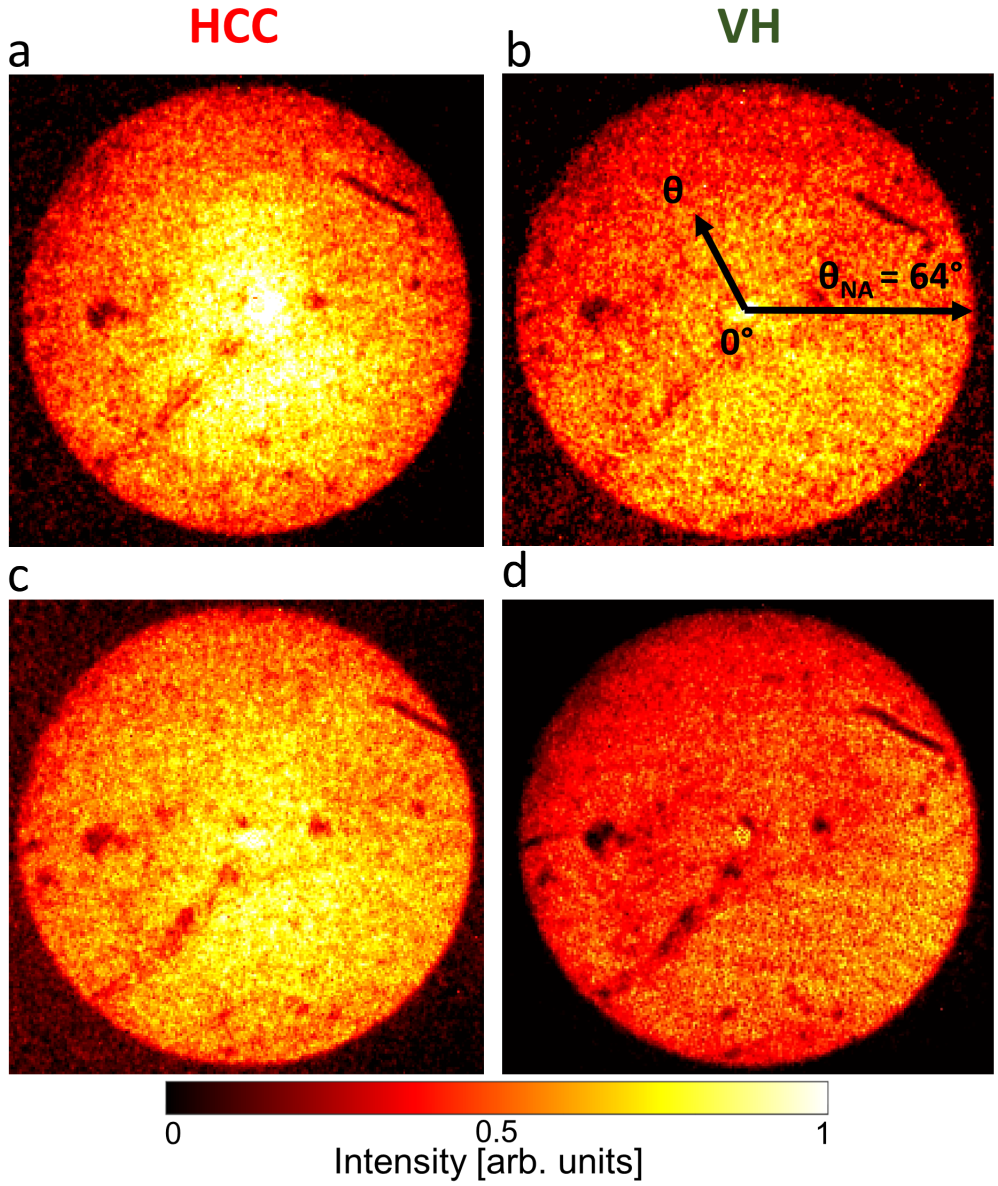

Figure S5: Extraction of the of the Raman Coherent Backscattering Cone. Momentum-space images of the nanowire Raman signal acquired in (a,c) the helicity conserving channel (HCC) portraying the superposition of both coherent signal and incoherent background, and (b,d) the linearly cross-polarized channel (VH) exclusively portraying the incoherent background for Sample 1 (a,b) and 2 (c,d), respectively. For each sample, the Raman coherent backscattering cone is obtained by processing the average intensity profiles extracted from these images (Figure 4 and Supplementary Section S5). The background-subtracted images are shown normalized to the same maximum intensity for the comparison. The positive angle θ sketched in b represents the angular cone of acceptance between 0° and θ_{NA} = 64° (i.e. the angle defined by the numerical aperture of the objective) for all the images shown.

References

[1] B. Fazio, P. Artoni, M. A. Iatì, C. D'Andrea, M. J. Lo Faro, S. Del Sorbo, S. Pirotta, P. G. Gucciardi, P. Musumeci, C. S. Vasi, R. Saija, M. Galli, F. Priolo, A. Irrera, *Light Sci. Appl.* **2016**, *5* (4), e16062–e16062.

[2] M. J. Lo Faro, A. A. Leonardi, F. Priolo, B. Fazio, M. Miritello, Irrera, A. *Sci. Rep.* **2020**, *10* (1), 12854.

[3] M. J. Lo Faro, A. A. Leonardi, C. D'Andrea, D. Morganti, P. Musumeci, C. Vasi, F. Priolo, B. Fazio, A. Irrera, *J. Mater. Sci. Mater. Electron.* **2020**, *31* (1), 34–40.

[4] "FracLac for ImageJ" https://imagej.nih.gov/ij/plugins/fraclac/FLHelp/Introduction.htm.

[5] D. A. G. Bruggeman, *Ann. Phys.* **1935**, *416* (7), 636–664.

[6] A. Garahan, L. Pilona, J. Yin, *J. Appl. Phys.* **2007,** 101, 014320

[7] J. W. Goodman, *J. Opt. Soc. Am.* **1976**, *66* (11), 1145.
